# PTEN and P-4E-BP1 might be associated with postoperative recurrence of rectal cancer patients undergoing concurrent radiochemotherapy

**DOI:** 10.1186/s12885-024-12339-x

**Published:** 2024-05-13

**Authors:** Heng Zhang, Xiaofan Li, Wanjun Sun, Haoren Qin, Haipeng Li, Hao Yan, Huaqing Wang, Xipeng Zhang, Shiwu Zhang, Hui Wang

**Affiliations:** 1 Department of Oncology, Tianjin Union Medical Center, Nankai University, No.190 Jieyuan Road, Hongqiao District, Tianjin, 300121 P. R. China; 2 Department of Oncology, People’s Hospital of Rongcheng, Shandong Rongcheng, P. R. China; 3 Department of Colorectal Surgery, Tianjin Union Medical Center, Nankai University, Tianjin, P. R. China; 4 Department of Pathology, Tianjin Union Medical Center, Nankai University, Tianjin, P. R. China

**Keywords:** Rectal cancer, Concurrent radiochemotherapy, Local recurrence, PTEN, 4E-BP1

## Abstract

**Background:**

Local recurrence after surgery and radiochemotherapy seriously affects the prognosis of locally advanced rectal cancer (LARC) patients. Studies on molecular markers related to the radiochemotherapy sensitivity of cancers have been widely carried out, which might provide valued information for clinicians to carry out individual treatment.

**Aim:**

To find potential biomarkers of tumors for predicting postoperative recurrence.

**Methods:**

In this study, LARC patients undergoing surgery and concurrent radiochemotherapy were enrolled. We focused on clinicopathological factors and PTEN, SIRT1, p-4E-BP1, and pS6 protein expression assessed by immunohistochemistry in 73 rectal cancer patients with local recurrence and 76 patients without local recurrence.

**Results:**

The expression of PTEN was higher, while the expression of p-4E-BP1 was lower in patients without local recurrence than in patients with local recurrence. Moreover, TNM stage, lymphatic vessel invasion (LVI), PTEN and p-4E-BP1 might be independent risk factors for local recurrence after LARC surgery combined with concurrent radiochemotherapy.

**Conclusions:**

This study suggests that PTEN and p-4E-BP1 might be potential biomarkers for prognostic prediction and therapeutic targets for LARC.

**Supplementary Information:**

The online version contains supplementary material available at 10.1186/s12885-024-12339-x.

## Background

In recent years, the incidence of colorectal cancer (CRC) in China has been increasing continuously, and it has become the fifth most fatal malignant tumor [1]. As a number of studies have confirmed that concurrent radiochemotherapy can reduce local recurrence after rectal cancer surgery, adjuvant/neoadjuvant radiochemotherapy has become a standard treatment for stage II/III rectal cancer in China [2–4]. However, the rate of local recurrence in these patients remains at approximately 6.6–13.6% [5], which seriously affects the prognosis.

Previous studies showed that some clinicopathological features were associated with postoperative recurrence of rectal cancer [6, 7]. Recently, studies on molecular markers related to radiochemotherapy sensitivity of cancers have been widely carried out [8–11], which might play a positive role in ameliorating the implementation of the ‘watch and wait’ strategy after neoadjuvant radiochemotherapy or promoting accurate implementation of adjuvant radiochemotherapy for locally advanced rectal cancer (LARC) patients.

Mammalian target of rapamycin (mTOR), a serine/threonine kinase, has regulatory effects on cell proliferation, DNA damage repair, cell apoptosis and autophagy [12, 13], which is regulated by the PI3K-Akt axis and negatively regulated by PTEN and SIRT1 [14–16]. Moreover, 4E-BP1 and S6RP are the main downstream target proteins of mTOR. Previously, we carried out laboratory studies on the function and regulatory factors of mTOR. It was found that ionizing radiation could induce sustained activation of mTOR and promote the levels of pS6, p-4E-BP1 and p-Akt473 in rectal adenocarcinoma cells (not published). However, inhibition of mTOR could prolong the G0/G1 phase of the cell cycle and reduce DNA damage repair efficiency via attenuation of homologous recombination, which suggests that mTOR might play important roles in the radiosensitivity of rectal adenocarcinoma cells.

In this study, LARC patients who underwent surgery and preoperative/postoperative radiochemotherapy at Tianjin Union Medical Center, Nankai University were enrolled, and a retrospective clinical study was carried out. We investigated clinicopathological features and expression of mTOR-related proteins in patients with or without local recurrence and explored the risk factors for local recurrence of LARC after surgery.

## Methods

### Patients

The clinical data of 1599 rectal cancer patients who received radiotherapy with concurrent 5-fluorouracil (5-FU) based chemotherapy and total mesorectal excision (TME) surgery were collected from January 2012 through May 2020, from Tianjin Union Medical Center, Nankai University. Clinical staging was classified by endosonography, computed tomography (CT) scan, and magnetic resonance imaging (MRI). The inclusion criteria were as follows; (1) pathologically diagnosed rectal adenocarcinoma; (2) cT3, cT4 or N + according to American Joint Committee on Cancer (AJCC) criteria (7th edition); (3) no evidence of distant metastasis; and (4) Karnofsky performance status over 70. To perform the study, 109 recurrent patients were selected, and another 109 patients were randomly selected from 1490 non recurrent ones. Sixty-nine patients with incomplete pathologic data or follow up data were excluded, reducing the total number of patients analyzed to 149, which were divided into a local recurrence group (LR, *n* = 73) and a nonrecurrence group (NR, *n* = 76).

### Treatment

All patients underwent radiotherapy with conventional fractionation of 1.8 Gy up to a total dose of 45 Gy in 25 fractions. For patients undergoing preoperative radiotherapy, an additional 5.4 Gy in 3 fractions was delivered as a boost to the gross tumor volume (GTV). The clinical target volume (CTV) was defined as the GTV and regional lymphatics including the mesorectal, presacral, internal iliac, and distant common iliac lymph nodes. To cover the CTV properly, the borders of the pelvic fields were as follows: (1) superior: the sacral promontory at the L5-S1 junction level, (2) inferior: 3 to 5 cm distal to the GTV, (3) lateral: 1.5 cm lateral to the widest bony margin of the true pelvic walls, (4) anterior: posterior border of the symphysis pubis, and (5) posterior: 1.5 cm behind the anterior bony sacral margin. The concurrent chemotherapy regimens were as follows: (1) CAPEOX, two cycles of L-OHP (130 mg/m^2^ day1) and capecitabine (825 mg/m^2^ twice daily day1-14 ) given at the first, and fourth weeks of radiotherapy; (2) CAPE, oral capecitabine (825 mg/m^2^ twice daily day1-5) given at every week of radiotherapy; (3) mFOLFOX6, L-OHP (85 mg/m^2^ day1), calcium levofolinate (200 mg/m^2^ day1) and 5-Fu (400 mg/m^2^ bolus, 2400 mg/m^2^ civ 46 h) given at the first, third and fifth weeks of radiotherapy; and (4) Tegafur (20 mg/kg day1-3) given at the first, and fourth weeks of radiotherapy. Patients receiving neoadjuvant therapy underwent planned TME surgery 4–6 weeks after the end of radiotherapy, and patients receiving adjuvant therapy underwent TME surgery 4–8 weeks before radiotherapy.

### Follow up

The patients were seen every 3 months during the first 2 years after surgery and every 6 months thereafter. Follow-up evaluations included physical examination, complete blood count, biochemical profile, abdominopelvic CT (or MRI), chest radiography, and proctoscopy (every 1 year).

### Tissue microarray (TMA) construction

Tissue samples were fixed in buffered 4% formalin, paraffin embedded, and used for TMA construction. Hematoxylin-eosin–stained histologic sections from resection specimens were used to mark representative tumor areas. A 1.0-mm tissue core was punched out from the tumor area of each specimen and transferred to a TMA recipient block using a TM-1 tissue arrayer (Bilcool Inc, Beijing, China). Three TMAs contained 149 tumor spots consisting of 73 rectal cancers of the local recurrence group and 76 rectal cancers of the nonrecurrence group.

### Immunohistochemical staining

The 4-µm–thick sections from TMA were deparaffinized and rehydrated using xylene and ethanol, and immersed in a 3% hydrogen peroxide solution for 10 min to block endogenous peroxidase. The sections were then boiled for 30 min in 10 mM/L citrate buffer solution (pH 6.0) for antigen retrieval. Slides were incubated with 5% skim milk for 20 min and then with PTEN antibody (1:1000, #9559, Cell Signaling Technology, Danvers, MA, USA), SIRT1 antibody (1:2000, ab110304, Abcam Biotechnology, Cambridge, MA, UK), p-4E-BP1 antibody (1:1000, #2855, Cell Signaling Technology, Danvers, MA, US) and pS6 antibody (1:1000, #5364, Cell Signaling Technology, Danvers, MA, USA) at 4 °C for 16 h, and visualized using the SP-9000 Polymer Detection System (Golden Bridge International Corp, Wharton, TX, USA) following the manufacturer’s instructions. Subsequently, the slides were incubated with 3, 3′-diaminobenzidine tetrahydrochloride-H_2_O_2_ solution for visualization, and counterstained with hematoxylin. All slices were observed under an Olympus DS-Ri2 microscope (Olympus, Tokyo, Japan), and PTEN, SIRT1, p-4E-BP1, and pS6 staining was defined as yellow or brown particles in either the nucleus or the cytoplasm. Immunostained slides were evaluated by two experienced pathologists who were blinded to all clinical parameters. The staining results were evaluated using a semiquantitative method: the percentage of stained cells × the staining intensity. This percentage was scored as 0 for < 5%, 1 for 5–25%, 2 for 26–50%, 3 for 51–75%, and 4 for 76–100%, and the staining intensity was then scored as 0 for no staining, 1 for light yellow, 2 for yellow brown, and 3 for brown. A score less than 4 is defined as negative expression, and a score greater than or equal to 4 is defined as positive expression [17].

### Statistical analysis

The Chi-square test or Fisher’s exact test was used for categorical variables. Logistic regression analysis was applied to examine the factors associated with local recurrence. A *P* value of < 0.05 was considered statistically significant. All statistical analyses were performed using SPSS version 26.0 (IBM Inc., Chicago, IL, USA).

## Results

### Patient characteristics

We studied 149 rectal cancer patients, comprising 73 LR cases (50 males, 23 females) and 76 NR cases (50 males, 26 females). The median age of these patients at diagnosis was 61.2 years in the LR group and 59.1 years in the NR group. There was no significant difference between the LR and NR group with the distance of tumor from the anal verge. Histologically, 78 (52.3%) tumors were classified as well or moderately differentiated carcinoma, 66 (44.3%) tumors were classified as poorly differentiated, and 5 (3.4%) were uncertain. Ninety-nine (66.4%) tumors were smaller than 5 cm, and 50 (33.6%) tumors had a minimum diameter of 5 cm or more. N stage was N0 for 36 (24.2%) tumors, N1 for 67 (45.0%) tumors and N2 for 46 (30.9%). Fifty-one (34.2%) cases were classified as stage II and 98 (65.8%) cases were classified as stage III according to the TNM staging system. Seventeen (11.4%) patients had lymphatic vessel invasion (LVI) and 132 (88.6%) patients did not. All patients underwent TME with a curative aim, in which 83 (55.7%) patients received Dixon surgery, 43 (28.9%) patients received Miles surgery, and 23 (15.4%) patients received Hartmann surgery. Seventy-three (49.0%) patients underwent neoadjuvant radiochemotherapy, and 76 (51.0%) patients underwent adjuvant radiochemotherapy. However, there were 8 (5.4%) patients whose circumferential resection margin was involved and 2 (1.3%) patients whose distal margin was involved. The concurrent chemotherapy regimens during radiotherapy were CAPE (53, 35.6%), CAPEOX (30, 20.1%), mFOLFOX6 (64, 43.0%), or Tegafur (2, 1.3%). As shown in Tables 1 and 2, and Supplementary Table 1, LNM, TNM stage and LVI might be correlated with local recurrence, while other characteristics were similar between LR and NR patients.

**Table 1:** Patient characteristics and clinicopathological data. (^*^ Fisher’s exact test)

Clinicopathological features			Total	LR *n* (%)	NR *n* (%)	χ²	*P*
			(*n* = 149)	73	76		
Gender							
	Male		97	50(68.5%)	50(65.8%)	0.123	0.725
	Female		52	23(31.5%)	26(34.2%)		
Age (y)							
	>50		134	64(87.7%)	70(92.1%)	0.809	0.369
	≤ 50		15	9(12.3%)	6(7.9%)		
Tumor location from anal verge (cm)							
	<5 cm		42	17(23.3%)	25(32.9%)	4.78	0.092
	5–10 cm		96	53(72.6%)	43(56.6%)		
	>10 cm		11	3(4.1%)	8(10.5%)		
Tumor size (cm)							
		<5 cm	99	48(65.8%)	51(67.1%)	0.031	0.861
		≥ 5 cm	50	25(34.2%)	25(32.9%)		
TNM stage							
		II	51	13(17.8%)	38(50.0%)	17.14	<0.001
		III	98	60(82.2%)	38(50.0%)		
T stage							
		T2	7	2(2.7%)	5(6.6%)	1.266	0.595^*^
		T3	117	58(79.5%)	59(77.6%)		
		T4	25	13(17.8%)	12(15.8%)		
N stage							
		N0	36	13(17.8%)	23(30.3%)	6.223	0.045
		N1	67	31(42.5%)	36(47.4%)		
		N2	46	29(39.7%)	17(22.3%)		
Differentiation							
		Well	4	3(4.1%)	1(1.3%)	2.592	0.490^*^
		Moderately	74	36(49.3%)	38(50.0%)		
		Poorly	66	33(45.2%)	33(43.4%)		
		Unknown	5	1(1.4%)	4(5.3%)		
Lymphatic vessel invasion							
		+	17	16(21.9%)	1(1.3%)	15.636	<0.001^*^
		-	132	57(78.1%)	75(98.7%)		
Circumferential resection margin							
		+	8	6(8.2%)	2(2.6%)	1.320	0.250^*^
		-	141	67(91.8%)	74(97.4%)		
Longitudinal resection margin							
		+	2	2(2.7%)	0(0%)	0.549	0.459^*^
		-	147	71(97.3%)	76(100%)		

**Table 2:** Correlations of treatment features with local recurrence of rectal cancer. (^*^ Fisher’s exact test)

Treatment		Total *n* = 149	LR *n* (%) 73	NR *n* (%) 76	χ²	*P*
Surgery						
	Dixon	83	34(46.6%)	49(64.5%)	5.51	0.064
	Miles	43	27(37.0%)	16(21.0%)		
	Hartmann	23	12(16.4%)	11(14.5%)		
Radiochemotherapy						
	neoadjuvant	73	40(54.8%)	33(43.4%)	1.927	0.165
	adjuvant	76	33(45.2%)	43(56.6%)		
Chemotherapy regimens						
	CAPE	53	29(39.7%)	24(31.6%)	1.664	0.736^*^
	CAPEOX	30	15(20.5%)	15(19.7%)		
	mFOLFOX6	64	28(38.4%)	36(47.4%)		
	Tegafur	2	1(1.4%)	1(1.3%)		

### PTEN and p-4E-BP1 might be associated with postoperative recurrence

**Fig. 1:**
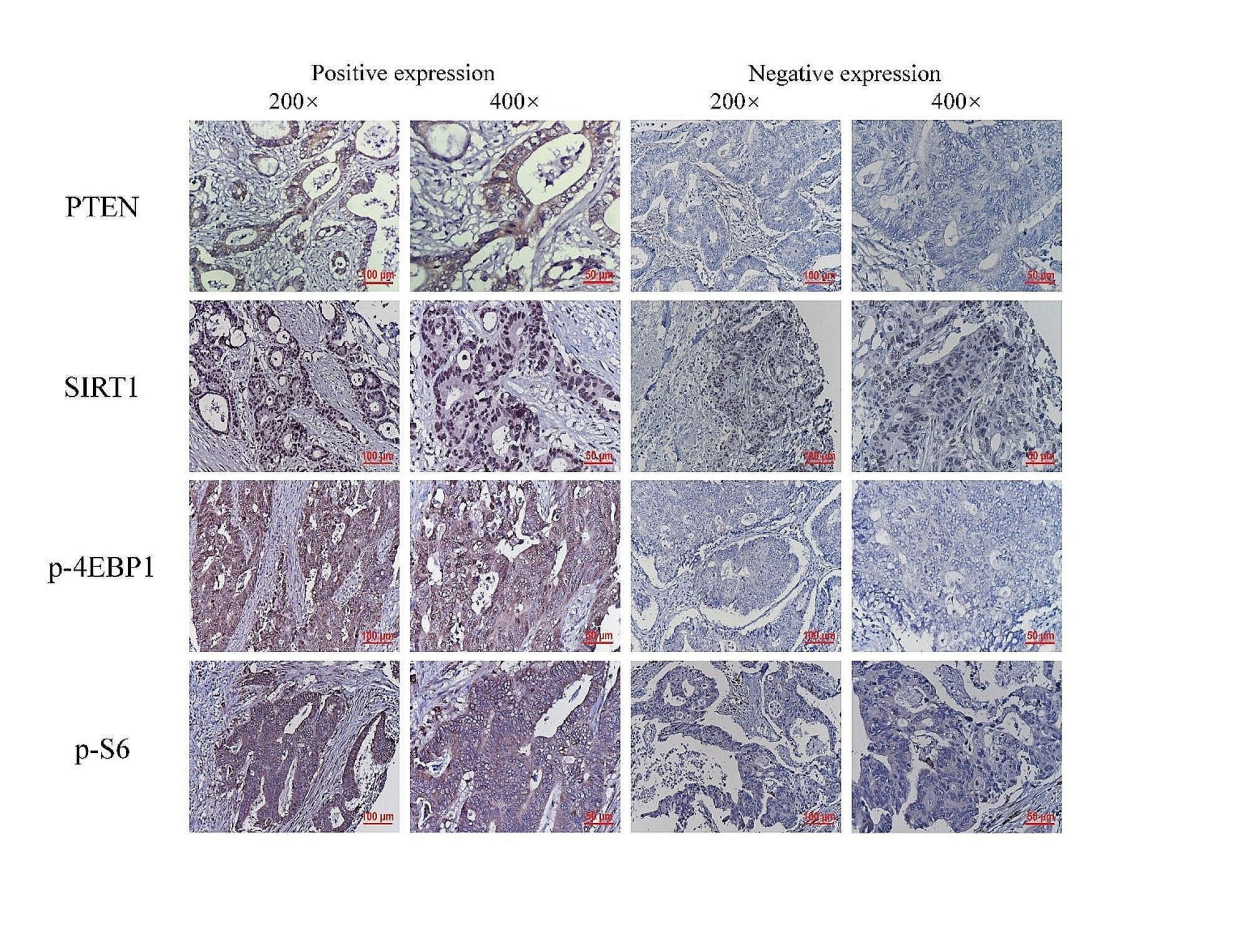
Representative immunohistochemical staining of PTEN, SIRT1, p-4E-BP1, and pS6 in rectal cancer patients

A total of 149 patient samples were subjected to immunohistochemical analysis in this study, and the results are shown in Fig. 1; Table 3. The PTEN protein, a negative regulator of mTOR, was mainly localized in the cytoplasm. The total positive rate of PTEN protein expression in all cases was 63.1% (94/149), and the positive rate in the LR group was 53.4% (39/73), which was lower than that in the NR group (72.4%, 55/76). The difference was statistically significant (*P* < 0.05). SIRT1 protein, mainly localized in the nucleus, can inhibit mTOR activity [16]. The total positive rate of SIRT1 protein expression in all patients was 83.2% (124/149), and there was no significant difference in SIRT1 expression between the LR group and the NR group (84.9% vs. 81.6%, *P* > 0.05). The p-4E-BP1 protein was mainly localized in the cytoplasm, and its expression level was positively correlated with mTOR activity. The total positive rate of p-4E-BP1 protein expression in all cases was 64.4% (96/149), of which 74.0% (54/73) vs. 55.3% (42/76) were in the LR group and NR group, respectively, and the difference was statistically significant (*P* < 0.05). PS6, another marker of mTOR activation protein, was mainly localized in the cytoplasm. The positive rate of pS6 protein expression in all patients was 79.2% (118/149), and there was no significant difference in pS6 expression between the LR group and the NR group (73.7% vs. 84.9%, *P* > 0.05).

**Table 3:** Comparisons of SIRT1, PTEN, p-4E-BP1, and pS6 protein expression in rectal cancer patients with and without local recurrence

	LR group *n* (%)	NR group *n* (%)	χ²	*P*
SIRT1				
-	11(15.1%)	14(18.4%)	0.300	0.584
+	62(84.9%)	62(81.6%)		
PTEN				
-	34(46.6%)	21(27.6%)	5.738	0.017
+	39(53.4%)	55(72.4%)		
p-4E-BP1				
-	19(26.0%)	34(44.7%)	5.687	0.017
+	54(74.0%)	42(55.3%)		
pS6				
-	11(15.1%)	20(26.3%)	2.859	0.091
+	62(84.9%)	56(73.7%)		

We included meaningful clinical, pathological and protein expression results in the logistic multivariate regression analysis. As shown in Table 4, the results showed that TNM stage, LVI, PTEN negativity and p-4E-BP1 positivity were all independent risk factors for local recurrence after TME surgery combined with concurrent radiochemotherapy (*P* < 0.05).

**Table 4:** Multivariate analysis of local recurrence

Covariates		Sig	OR	95% C.I.	
				Lower	Upper
TNM stage					
	II		1		
	III	<0.001	4.515	1.935	10.535
Lymphatic vessel invasion					
	Negative		1		
	Positive	0.006	24.97	2.557	243.815
PTEN					
	+		1		
	-	0.001	5.312	1.968	14.337
p-4E-BP1					
	-		1		
	+	0.003	4.402	1.635	11.857

## Discussion

In this study, we focused on SIRT1-related protein (PTEN, SIRT1, p-4E-BP1, and pS6) expression as assessed by immunohistochemistry in 73 rectal cancer patients with local recurrence and 76 patients without local recurrence after TME surgery and concurrent radiochemotherapy. The expression of PTEN was higher while the expression of p-4E-BP1 was lower in patients without local recurrence than in patients with local recurrence, suggesting that mTOR activation might be implicated in the local recurrence of rectal cancer.

Before the era of preoperative radiochemotherapy became the standard treatment, several prognostic factors for predicting recurrence were reported [18, 19], such as tumor size, pathological T and N stages, histologic grade, vascular invasion, and carcinoembryonic antigen (CEA) level. In recent years, a part of LARC patients with neoadjuvant radiochemotherapy obtained ideal tumor regression, while it is difficult to obtain comprehensive pathological information, such as vascular invasion, tissue differentiation, and pTNM stage [3, 5]. 

It is widely accepted that currently only clinical-pathological information will not be robust enough to have prognostic and predictive utility. Recently, molecular biomarkers, involved in cell proliferation, apoptosis, DNA damage repair, cell cycle, angiogenesis, and epithelial to mesenchymal transition have been widely investigated [20, 21]. These include tumor tissue biomarkers of either biopsy or surgical specimens, and blood-based biomarkers as well as tumor related nucleic acids and circulating tumor cells (CTCs). As yet, not a single marker is adequate to have clinical utility. Screening biomarkers of tumors by using microarrays would likely have higher yield and low cost, and it is expected that a combination of markers would prove most useful [22, 23]. Biopsy, blood, and CTCs could be obtained for checking biomarkers at different stages of patients’ diagnosis and treatment, which would be expected to provide valuable information for clinicians to fulfill individual treatment.

The PI3K/mTOR signaling pathway, modulating tumor initiation and development in many types of cancers, is a potential candidate to predict the response to radiochemotherapy [24–26]. The main regulator of this system is PTEN, which can stop signaling by removing 3’-phosphate groups. Therefore, the PI3K/mTOR pathway contributes to the phosphorylation of 4E-BP1, which results in the formation of the cap-dependent mRNA translation initiation complex [27], and the regulation of protein synthesis, cell growth and proliferation. In recent years, many scholars were concerned about the potential linkage between loss of PTEN and chemotherapy resistance to cetuximab in advanced colorectal cancer [28–34]. A few studies have discussed the relationship between PTEN and radiation sensitivity. Philipp’s study and Dellas’s study suggested PTEN expression do not predict efficacy of cetuximab-based radiochemotherapy in LARC [35, 36]. In Oya’ s work [37], basal mRNA expression level of PTEN has been investigated, and it was suggested PTEN could be a potential predictive elements for radiochemotherapy. Compared with PTEN, fewer works about 4E-BP1 were reported. Chen’s work [12] manifested that p-4E-BP1 might be a biomarker to predict chemotherapy outcome of patients with colorectal cancer, but not radiotherapy outcome. Our study showed that PTEN and p-4E-BP1 were expressed in LARC, and multivariate analysis in 169 patients demonstrated that the levels of PTEN and p-4E-BP1 were significantly associated with local recurrence after surgery combined with radiochemotherapy. Our results are in agreement with previous studies in esophageal squamous cell carcinoma, renal cell carcinoma, hilar cholangiocarcinoma, and lung cancer [38–41].

## Conclusion

The results of this study suggest that PTEN and p-4E-BP1 might be potential tumor markers for prognostic prediction and a therapeutic target for LARC. Although no predictive biomarkers for response to LARC are sufficiently robust to have clinical utility, the integration of diverse types of biomarkers, including clinicopathological and imaging features will allow the development of a sensitive molecular biomarker panel and enhance efforts for personalized care in rectal cancer patients.

### Electronic supplementary material

Below is the link to the electronic supplementary material.

Supplementary Material 1

Supplementary Material 2

## Data Availability

The data sets that support the conclusions of this article are included within the article.

## Abbreviations

LARC: locally advanced rectal cancer
LVI: lymphatic vessel invasion
CRC: colorectal cancer
mTOR: mammalian target of rapamycin
TME: total mesorectal excision
CT: computed tomography
MRI: magnetic resonance imaging
AJCC: American Joint Committee on Cancer
LR: local recurrence
NR: nonrecurrence
GTV: gross tumor volume
CTV: clinical target volume
TMA: tissue microarray
CTCs: circulating tumor cells
CEA: carcinoembryonic antigen
