# Supplementary material for: PTEN and P-4E-BP1 might be associated with postoperative recurrence of rectal cancer patients undergoing concurrent radiochemotherapy
Source: BMC Cancer. 2024 May 13;24:582. doi: 10.1186/s12885-024-12339-x (PMC11089754; doi:10.1186/s12885-024-12339-x)
Supplement: Supplementary file 1 — Supplementary Material 1 [file 12885_2024_12339_MOESM1_ESM.docx]

| Treatment | | Total n=149 | LR n (%)  73 | NR n (%)  76 | *χ²* | *P* |
| --- | --- | --- | --- | --- | --- | --- |
| CAPE | |  |  |  |  |  |
|  | neoadjuvant | 28 | 17(60.7%) | 11(39.3%) | 0.862 | 0.353 |
|  | adjuvant | 25 | 12(48.0%) | 13(52.0%) |  |  |
| CAPEOX | |  |  |  |  |  |
|  | neoadjuvant | 12 | 7(58.3%) | 5(41.7%) | 0.139 | 0.709* |
|  | adjuvant | 10 | 8(47.4%) | 10(52.6%) |  |  |
| mFOLFOX6 | |  |  |  |  |  |
|  | neoadjuvant | 33 | 16(48.5%) | 17(51.5%) | 0.621 | 0.431 |
|  | adjuvant | 31 | 12(36.7%) | 19(63.3%) |  |  |
| Tegafur | |  |  |  |  |  |
|  | neoadjuvant | 0 | 0 | 0 |  |  |
|  | adjuvant | 2 | 1 | 1 |  |  |

**Supplementary table 1.** Correlations of treatment features with local recurrence of rectal cancer. (* Fisher’s exact test)
