# Supplementary figures and images for: PTEN and P-4E-BP1 might be associated with postoperative recurrence of rectal cancer patients undergoing concurrent radiochemotherapy
Source: BMC Cancer. 2024 May 13;24:582. doi: 10.1186/s12885-024-12339-x (PMC11089754; doi:10.1186/s12885-024-12339-x)

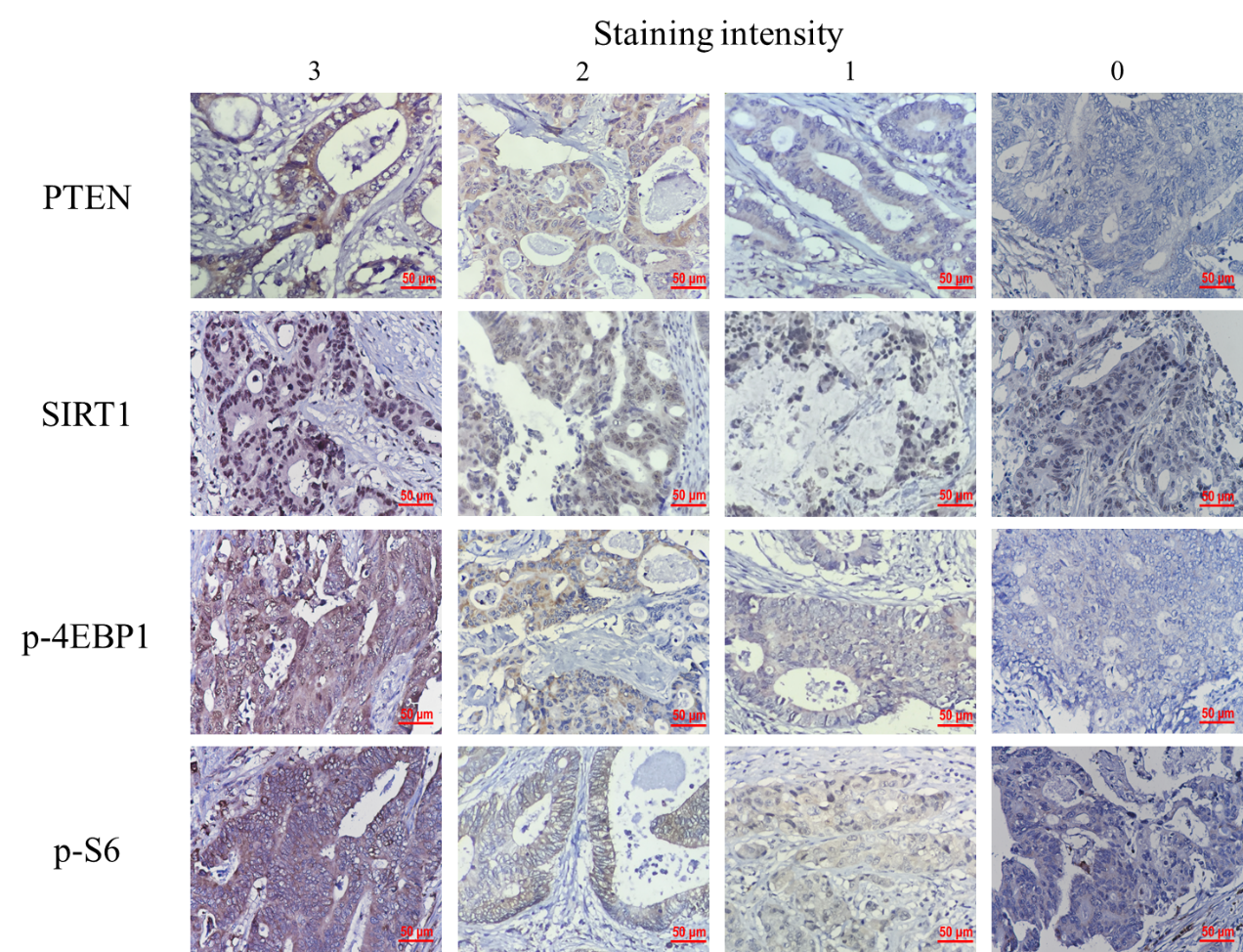


**Supplementary figure 1.** The intensity of staining with antibodies of PTEN, SIRT1, p-4E-BP1, and pS6.

Supplement: Supplementary file 2 — Supplementary Material 2 [file 12885_2024_12339_MOESM2_ESM.docx]
